# Public perspectives on increased data sharing in health research in the context of the 2023 National Institutes of Health Data Sharing Policy

**DOI:** 10.1371/journal.pone.0309161

**Published:** 2024-08-28

**Authors:** Stephanie Niño de Rivera, Ruth Masterson Creber, Yihong Zhao, Sarah Eslami, Sabrina Mangal, Lydia S. Dugdale, Meghan Reading Turchioe

**Affiliations:** 1 Columbia University School of Nursing, Columbia University Irving Medical Center, New York, New York, United States of America; 2 Biobehavioral Nursing & Health Informatics, University of Washington School of Nursing, Seattle, Washington, United States of America; 3 Center for Clinical Medical Ethics, Columbia University Vagelos College of Physicians & Surgeons, New York, New York, United States of America; University of KwaZulu-Natal College of Health Sciences, SOUTH AFRICA

## Abstract

The National Institutes of Health (NIH) is the largest public research funder in the world. In an effort to make publicly funded data more accessible, the NIH established a new Data Management and Sharing (DMS) Policy effective January 2023. Though the new policy was available for public comment, the patient perspective and the potential unintended consequences of the policy on patients’ willingness to participate in research have been underexplored. This study aimed to determine: (1) participant preferences about the types of data they are willing to share with external entities, and (2) participant perspectives regarding the updated 2023 NIH DMS policy. A cross-sectional, nationally representative online survey was conducted among 610 English-speaking US adults in March 2023 using Prolific. Overall, 50% of the sample identified as women, 13% as Black or African American, and 7% as Hispanic or Latino, with a mean age of 46 years. The majority of respondents (65%) agreed with the NIH policy, but racial differences were noted with a higher percentage (28%) of Black participants indicating a decrease in willingness to participate in research studies with the updated policy in place. Participants were more willing to share research data with healthcare providers, yet their preferences for data sharing varied depending on the type of data to be shared and the recipients. Participants were less willing to share sexual health and fertility data with health technology companies (41%) and public repositories (37%) compared to their healthcare providers (75%). The findings highlight the importance of adopting a transparent approach to data sharing that balances protecting patient autonomy with more open data sharing.

## Introduction

In January 2023, the National Institutes of Health (NIH) updated its Data Management and Sharing (DMS) policy to increase data sharing practices for the purposes of transparency and reproducibility [1]. The NIH provides the largest amount of public funding for research studies in health of any US federal agency, and the updated policy mandates NIH-funded researchers to make their datasets available in data repositories [2, 3]. Data repositories serve as platforms for researchers to access research data for secondary purposes [4] and include data collected in clinical trials, except for data covered by privacy law or excluded in initial participant consent forms.

Many of the datasets contain data from electronic health records (EHRs), claims, biobanks, and patient-reported outcomes, and include sensitive information, such as sexual health, fertility, and mental health data. Private companies may be among those accessing these datasets as they may have commercial value [5]. Repositories exhibit varying degrees of accessibility; some are open to the general public without oversight from ethics review boards while others enforce more stringent access policies [6, 7].

As a mechanism to facilitate increased data sharing, researchers can use broad consent for the secondary uses of research data [8]. At the time of consent, the future use of the data, including the industries that might have access to it, are not explicitly disclosed, as the potential future use is unknown [9]. The immediate and future consequences of the uncertainty around future access and use of data can compound mistrust in communities that are already more reluctant to participate in research [10–13]. Participants have previously expressed apprehension around data sharing in research, raising concerns about transparency and trust in research practices even before recent policy updates [10, 11]. For instance, in a narrative review of 27 papers exploring participant views towards research data sharing practices, Kalkman et al. found that participants are concerned about breaches of confidentiality and potential abuses of research data by external entities [13]. Therefore, it is imperative to ensure that research practices do not lead to unintended consequences, such as decreased trust which is already present in racial and ethnic minority communities that are underrepresented in research and less trustful of the research community due to historical abuse [14].

Although the public plays a crucial role as both contributors and consumers of health data, their perspectives regarding data sharing preferences for different types of data and attitudes toward recent NIH policy developments have not been well explored. Thus, this study aims to provide insight into public perspectives on those developments.

## Methods

### Ethics statement

The Institutional Review Board at Columbia University approved this study. Participants were administered an information sheet about the study and provided informed consent by checking a box on the online survey.

### Study design

In March 2023, a U.S. representative sample of 610 adults was recruited using Prolific, an online survey recruitment platform [14]. The recruitment platform consists of verified users willing to participate in research studies and facilitates a matching process between researchers and users for rapid recruitment. Prolific uses U.S. Census Bureau data to divide the sample into subgroups by age, gender, and race with the same proportions as the national population. A representative sample option was selected for the survey on the platform. Prolific stratifies age using seven brackets: 18–24, 25–34, 35–44, 45–54, 55–64, 65–74 and 74+. ‘Sex’ is stratified into male and female, while race adheres to the five categories outlined by the UK Office of National Statistics: White, Mixed, Asian, Black, and Other. Also, participants must reside in the country being surveyed and demonstrate fluency in its primary language. The sampling frame for our study encompassed all 50 U.S. states. Prolific sent out invitations to potential participants that met eligibility criteria to participate in the survey. The data were collected and stored using Qualtrics, a HIPAA-compliant survey development tool. Participants were compensated $15 per hour and were prorated according to time of completion.

The questions for this online survey were developed through a literature review and expert input from physicians, bioethicists, and nurse-scientists. The survey was pilot tested with 10 members of the general public using Prolific for clarity and length and then revised accordingly. This cross-sectional survey was conducted in English and collected sociodemographic characteristics and attitudes toward data sharing across four domains: (1a) recipients of identifiable research data, (1b) recipients of de-identified research data, (1c) specific data types, and (2) reactions to the NIH DMS policy. It collected primarily closed-ended quantitative items but also included a small number of open-ended qualitative items. The survey had different blocks so that participants would only see some questions at a time and have the relevant definitions needed for the questions that followed. For example, the definition of the NIH DMS policy was presented with only the questions that were related to the policy (e.g., “Do you agree or disagree with the NIH’s new efforts to make research data collected about you more accessible to the scientific community and public?”). The survey questions can be found in S1 File.

### Data collection

After logging in, participants first saw an overview of the study and were then asked to provide informed consent. Once they agreed to participate, they were asked about the groups (chosen family, chosen friends, doctors and nurses, or other healthcare providers) with whom they would be willing to share *identifiable* research data. Identifiable data were a separate category to clarify to participants that this data could be traced back and could be clinically meaningful for them (e.g., specific research results could be used by their healthcare team to inform their care). Second, they were asked about the external groups (health technology companies, public health organizations, health policy institutions, private foundations, or public platforms) with whom they would share *de-identified* or *aggregated* data. To ensure clarity, we provided examples of de-identified and aggregate data within the survey. Third, participants were informed about the potential for secondary uses of research data. They were then asked to specify their responses to sharing seven specific types of data that aligned with the NIH DMS policy (sexual health and fertility, mental health, genetic, imaging, biological, clinical, and consumer-generated data). Finally, participants were given a summary of the updated NIH DMS policy using lay terms and asked to express their opinions about the policy, with an option for open-text feedback.

### Statistical analysis

Descriptive statistics on sociodemographic variables and closed-ended survey responses were generated for the overall sample. Differences in responses by self-reported race were assessed with Pearson’s chi-squared test, and Fisher’s exact test was used in the cases of small sample cells. A secondary analysis examined differences by race.

### Qualitative analysis

We conducted a general thematic analysis of the open-ended survey responses about the NIH DMS policy. Two members of the research team concurrently reviewed a subset of responses and generated a preliminary list of themes. One researcher coded the remaining responses independently. The second researcher reviewed the final list of themes and illustrative quotes and discussed them with the second researcher until they reached a consensus.

## Results

### Sample characteristics

Participant sociodemographic characteristics are summarized in Table 1. Among 610 participants, 50% were female, with an average age of 46 years (standard deviation 16). Overall, 79% of participants self-identified as White, and 7% self-identified as Hispanic or Latinx. This closely matched with the US census data with respect to race [15]. The sample included participants from 46 US states and the District of Columbia (S1 Table). Over half of the participants completed a college degree or higher, and 31% of participants reported financial instability, indicated by the answer “not enough” financial resources. The average time of completion for the survey was 13 min and 55 seconds. There were two survey responses that took less than 5 minutes to complete, but they were not dropped after checking the quality of their responses.

**Table 1:** Demographic characteristics of the survey sample.

Variable	Overall (N = 610)	US Census Data
**Age** (mean, SD)	46 (16)	––
**Gender** (*%*)		
Female	50%	50.4%
Male	48%	47.2%
Nonbinary or gender diverse	2%	2.3%
**Race** (*%*)		
American Indian or Alaska Native	0.5%	1.3%
Asian	5.9%	6.3%
Black or African American	12.8%	13.6%
Native Hawaiian or Pacific Islander	0.2%	0.3%
White	77.9%	75.5%
Multirace	1.8%	3%
Identifies as a race not listed	1.0%	––
**Hispanic or Latino Origin** (*%*)	7%	19.1%
**Education** (*%*)		
High school graduate or less	14%	––
Some college or Bachelor’s	69%	––
Graduate degree	17%	––
**Financial resources** (*%*)		
Not enough	31%	––
Enough	57%	––
More than enough	12%	––

### Quantitative results

**Fig 1:**
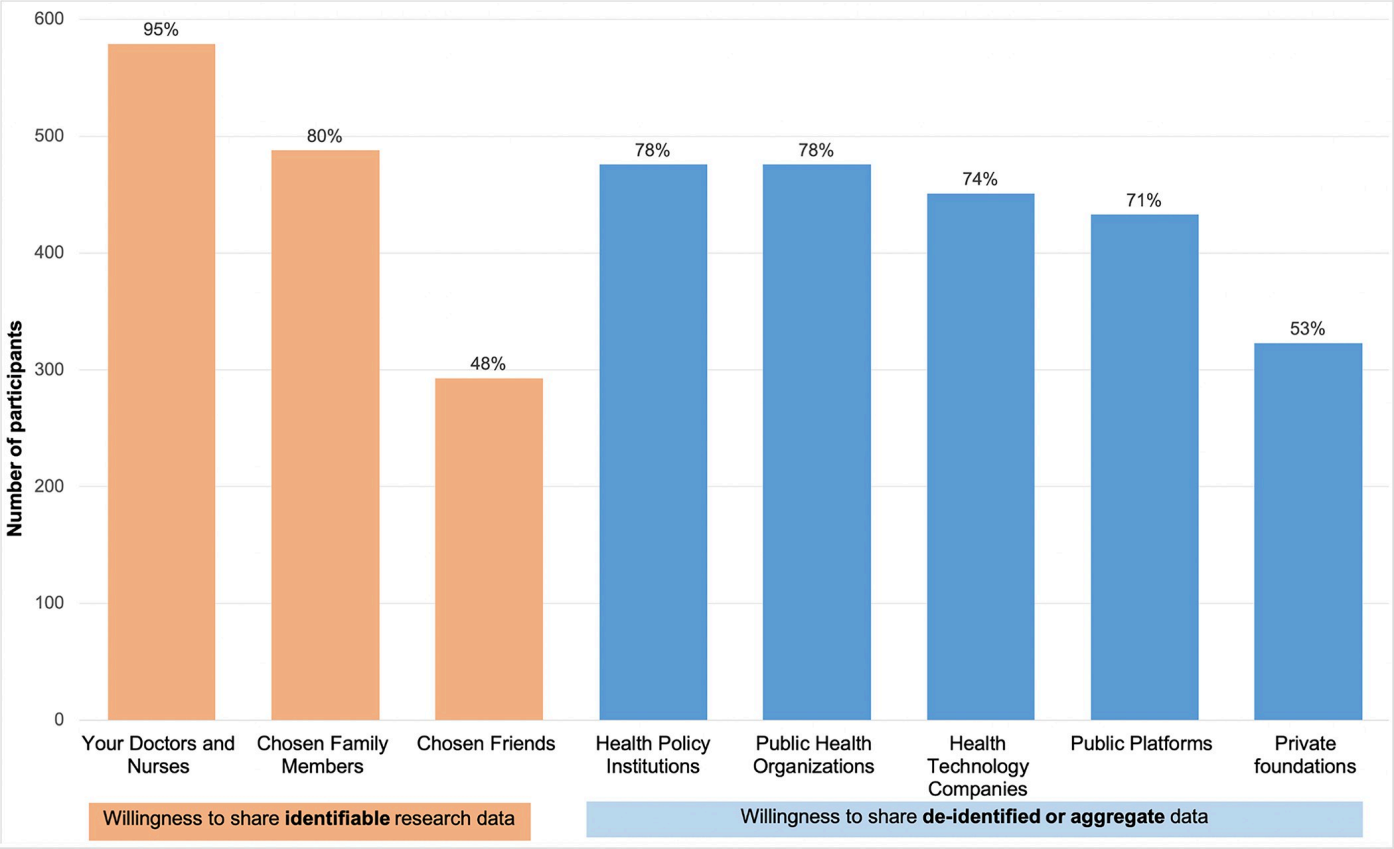
Participants’ willingness to share any research data with the following groups.

Data sharing preferences varied based on the recipient involved. The majority of participants (95%) were willing to share identifiable research data with doctors and nurses, but fewer (48%) would share it with chosen friends (Fig 1). Most participants (71–78%) were willing to share de-identified data with most external groups, including health technology companies, but fewer (53%) would share it with private foundations.

**Fig 2:**
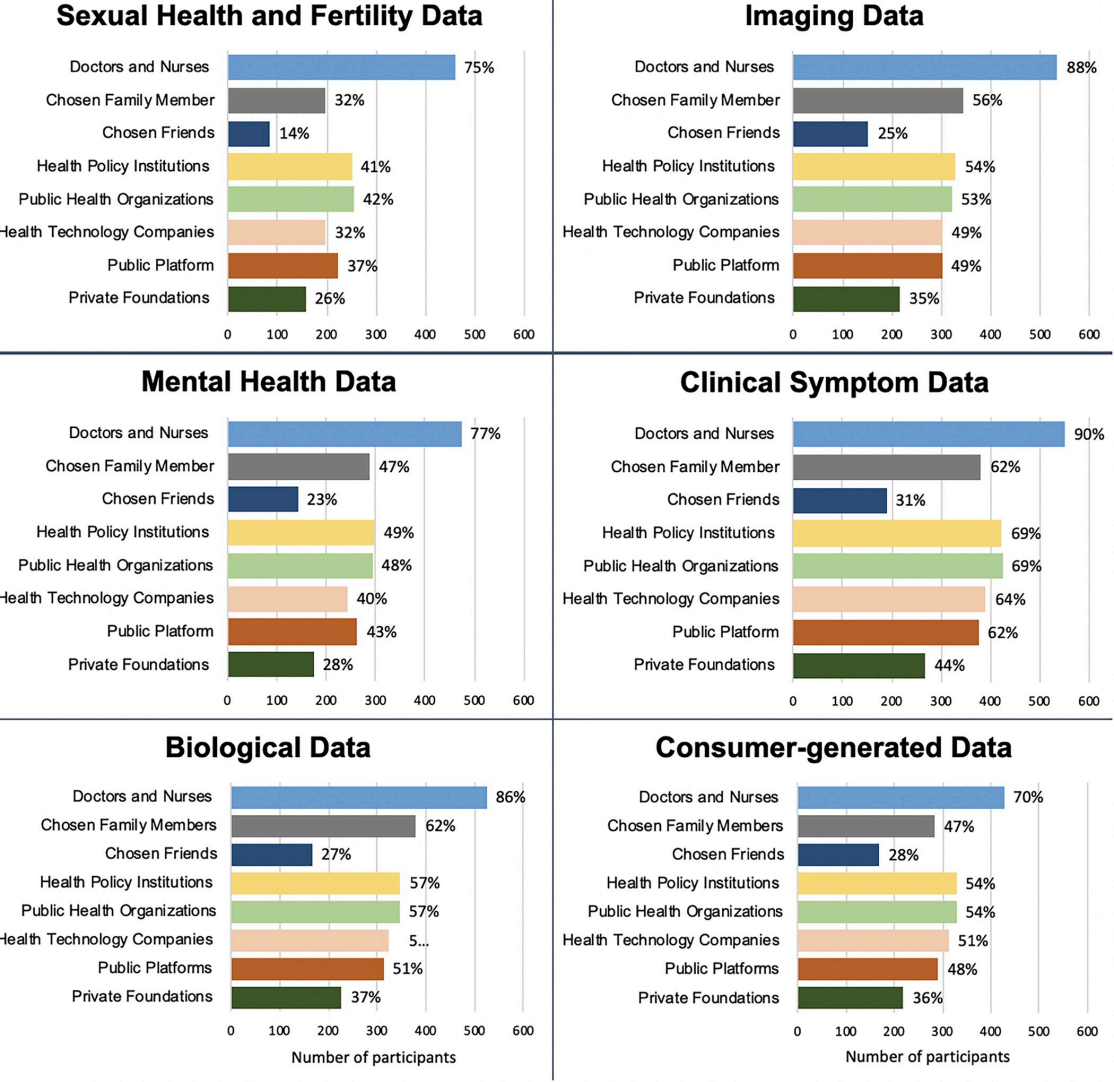
Specific types of research data participants want to share with various external groups.

Participants’ preferences varied based on the type of data. Across all types of data, participants were most willing to share their data with doctors and nurses (Fig 2). The smallest proportion of participants were willing to share their sexual health and fertility data across all the external recipients (with the exception of doctors and nurses): chosen family member (32%), chosen friends (14%), health policy institution (41%), health technology companies (32%), public platform (37%). Similarly, less than 50% of participants indicated a willingness to share mental health data with external recipients outside of their healthcare team. Many participants were more willing to share genetic data with family members (68%) and healthcare professionals (81%), but less willing to share with private foundations (31%) and health technology companies (44%). Furthermore, of participants who decided to share consumer-generated data, 70% chose doctors and nurses, 51% health technology companies, 54% health policy institutions, and 48% public platforms. The entire analysis on the different types of data can be found in Fig 2.

Regarding perspectives on the 2023 NIH DMS Policy (Table 2), more than half (65%) of participants either agreed or strongly agreed with it, but 17% disagreed or strongly disagreed. The policy would not change more than half (61%) of participant’s willingness to participate in research. There were significant differences in perspectives by race; a higher percentage of Black/African American participants (23%) disagreed or strongly disagreed with the policy compared to all other racial groups. Additionally, a higher percentage of Black participants (28%) indicated a decrease or strong decrease in their willingness to participate in research studies in response to the updated policy compared to most other racial groups.

**Table 2:** Survey questions regarding the updated NIH DMS policy and perceived impacts.

	Overall (N = 610)	White (n = 475)	Black/ African American (n = 78)	Asian (n = 36)	Identified with another race* (n = 21)	P-value
**Q1.** Do you agree or disagree with the NIH’s new efforts to make research data collected about you more accessible to the scientific community and the public?						
Strongly agree or agree	65%	67%	50%	72%	86%	**<0.01**
Neither agree or disagree	18%	16%	27%	19%	14%	
Strongly disagree or disagree	17%	17%	23%	9%	0%	
**Q2**. Does the NIH’s new efforts change your willingness to participate in a research study?						
Strongly increase or increase	23%	22%	26%	25%	0%	**<0.01**
No change	61%	63%	46%	64%	67%	
Strongly decrease or decrease	16%	15%	28%	11%	33%	

*Identified with another race included: American Indian or Alaska Native, Native Hawaiian or Pacific Islander, Multirace, or Other specification

### Qualitative analysis results

In response to the question regarding the new NIH Data Management and Sharing Policy, 302 (49%) participants provided written comments. The responses were categorized into one of the following themes: supportive of policy (33%), supported limiting access to data (37%), prioritized anonymity (17%), prioritized autonomy (6%), prioritized transparency (22%). Eight percent of responses were not categorized due to a theme not being applicable or identified. Some participants supported the policy, appreciating its potential benefits, such as transparency and the acceleration of research. However, many revealed concerns about the policy relating to a loss of autonomy, fear of misusing research data, lack of transparency, and lack of anonymity. Regarding concerns with loss of autonomy, participants felt that they were the rightful custodians of their data and should have control over designating data recipients, with one asserting, “It is my information, and I should be in charge of where it goes and who can access it.” (White female, 29) Other participants emphasized the importance of transparency in future uses of the data, as one mentioned, “While I agree that it can help to make positive changes, I would 100% want to know which outside companies they’re talking about before I would be willing…it does decrease my willingness [to participate].” (White female, 54) Finally, many participants were aware of how difficult it is to truly anonymize data, which also generated significant concerns: “By combining information from multiple sources, it is possible to de-anonymize data. If you know my age, zip code, and income, you are a long way towards knowing who I am.” (White Male, 66) These key themes are outlined with exemplary quotes in Table 3.

**Table 3:** Participants responses regarding the updated NIH Data Management and Sharing Policy*.

Emergent theme	Exemplary Quote
**Supportive of Policy**	“I like the idea of data sets being useful to more than one study and accelerating research.” (Asian female, 35)
**Supported Limiting Access to Data**	“While I agree that it can help to make positive changes, I would 100% want to know which outside companies they’re talking about before I would be willing. . .it does decrease my willingness.” (White male, 52)
**Prioritized Autonomy**	“I think the research participant should have a choice as to whom data would be shared.” (White female, 60)
	“It is my information and I should be in charge of where it goes and who can access it.” (White female, 29)
	“Though I completely understand the positives of what making information public would bring, people still deserve the right to choose to have that type of [data] private.” (Black male, 30)
**Prioritized Anonymity**	“All the policies in the world are great but as I’ve learned in doing cyber security, there is no safe system, there is no completely safe data.” (Multi-race male, 36)
	“By combining information from multiple sources, it is possible to de-anonymize data. If you know my age, zip code and income, you are a long way towards knowing who I am.” (White male, 66)
	Does the NIH guarantee that the data will be anonymized? Is the data ever sold or traded for goods and services?” (Black female, 61)
**Prioritized Transparency**	It seems logical. My only hesitation is influenced by being jaded by tech companies’ rampant data selling and sharing that I view as unethical.” (White male, 34)
	“I suppose it’s fine, I mostly don’t want private corps [corporations] to profit from it.” (White male, 49)
	“I would need to know exactly which public groups would have access. Neighbors? Insurance companies?” (White female, 49)

*Respondents answered the following open-text response: Provide any comments you have about the new NIH Data Management and Sharing Policy.

## Discussion

Participants’ willingness to share research data depends on the recipient group and the type of data involved. Variations in the types of data participants are willing to share with external entities highlights the need to evaluate broad data sharing practices in research. Furthermore, our findings reflect public perspectives considering the current research landscape, which is moving towards increased, broad data sharing practices.

Our present study reinforces participant concerns previously described prior to the 2023 NIH Data Sharing Policy. In alignment with previous literature, participant concerns such as the commercial use of research data, re-identification of de-identified data, and trust in research practices also emerged [10–13, 16]. Consequently, the research community must find solutions to develop data sharing policies that comprehensively address participants’ concerns to reinforce transparency and mitigate apprehension about participating in research studies. These findings have important implications for researchers who collect a broad range of datasets in their work, many of whom receive NIH funding and need to comply with the updated policy.

We found that participants were more willing to share all types of data with healthcare providers compared to other groups such as health technology companies or private foundations. Perceptions of how that data will be used beyond immediate patient care or research purposes may influence participants’ reluctance to share data with external recipients beyond the healthcare entities. Notably, we found that consumer-generated data were less likely to be shared with health technology companies compared to healthcare providers. This may be due to concerns previously raised about potential profit generation from health data used to develop new technologies [17, 18]. Our qualitative findings align with these sentiments, emphasizing concerns about others potentially profiting from participants’ health data. Previous studies focused on the collection of consumer-generated data, such as data from health apps, also shared these concerns regarding possible commercial agendas by external entities [13, 19, 20]. Mangal et al. also describes participants’ concerns about data commercialization and highlights how countries like Canada are addressing these issues through initiatives such as digital service taxation, which provides revenue back to communities [11].

One potential reason for reticence to share data in certain contexts, especially sensitive data such as sexual health and fertility data, could be due to concerns about re-identification. For instance, Chikwetu and colleagues highlight that despite efforts to anonymize data, various reidentification methods pose significant risks to privacy, especially in the context of wearable devices that individuals commonly use to track their health (e.g., reproductive health tracking, etc.) [21]. Furthermore, in our study, we found that, sexual health and fertility data are least likely to be shared with external entities beyond healthcare professionals, such as health technology companies and health policy institutions. This reluctance can be attributed to the growing sensitivity surrounding reproductive health issues in the United States, especially after the overturn of *Roe v*. *Wade* [22, 23]. These concerns center around limited reproductive rights and fears of data collection and sharing from fertility tracking applications, with apprehensions that such data could be used to prosecute participants for crimes and to track miscarriages or abortions [23]. Consequently, the exposure of these types of data through public repositories poses significant risks, especially for individuals subject to evolving laws, potentially making both the patients and their healthcare professionals criminally liable [24]. A similar pattern was observed for mental health data, with fewer participants willing to share it with external entities not directly involved in their care, primarily due to the stigma associated with mental health conditions [25, 26].

As the NIH and human sciences research moves toward enhancing research data accessibility, it is crucial to maintain a balance that also safeguards patient privacy and autonomy. While many participants displayed optimism toward the 2023 NIH DMS policy, individuals from underrepresented racial backgrounds expressed apprehension, which may be impacted by their knowledge of the history of unethical research studies and their experiences with ongoing systemic inequities in terms of access to healthcare and research today [27].

Previous research has highlighted the significant influence of data sharing practices on participants’ trust in the medical research community [28]. Trust in research can erode when participants feel they have limited control over how and with whom their personal health data are shared [10–12]. The unintended exposure of sensitive data through public repositories can inadvertently deter patients from participating in medical research or even seeking care at academic medical centers, where secondary use of medical records for research purposes may subject those records to the NIH data sharing policies [22]. Consequently, further research is warranted to address concerns related to broadly consenting to data sharing, participant autonomy in choosing where data are shared, and transparency regarding intentions of data use.

Our findings demonstrate that participants have varying levels of comfort regarding the sharing of specific types of data with certain groups. This supports previous findings of researchers moving towards implementing a participant-centered approach that offers customization options, allowing individuals to specify which data can be shared and with which industries and organizations [29]. The informed consent process presents an avenue through which willingness to participate in future research studies can be enhanced, ensuring that participant autonomy is upheld. Aside from participants having a greater influence on how their data is shared, the research community must also identify and promote best practices that facilitate increased regulation over the secondary uses of data. This can help participants feel comfortable to opt-in to share their data, specifically in the era of big data and AI-based algorithms and data analyses. AI by definition requires large amounts of data for training and testing, and the complexities of participant data-sharing preferences and the ethics of their data being used in these contexts are nuanced. Future work can examine if patient preferences differ when data are shared for building AI models. Moreover, it is imperative for researchers to prioritize the development of strategies that foster trust in data sharing practices within research. This requires researchers to ensure that participants have a comprehensive understanding of current practices to provide more details regarding the protection of data, especially among individuals who will be participating in a research study for their first time. By doing so, participants are empowered to make informed decisions in collaboration with researchers and researchers strengthen transparency of current data sharing practices.

## Limitations

Study limitations include the online and English-only survey that was conducted through the Prolific survey platform. There are limitations with the information we can access regarding the survey’s administration. Prolific does not provide information on the response rate of the individuals that invitations were sent out to participate in our study. In addition, to use Prolific, respondents must have familiarity with technology, access to the Internet, and English proficiency. These participants are also likely to have higher trust in researchers since they are actively seeking to participate in survey research studies. A limitation in our survey questions could include participants misinterpreting certain terms, such as private foundation, or having differing baseline levels in understanding privacy and security research measures around data sharing. Our race-related results are also constrained by relatively small subsamples of non-white participants. Due to sample size limitations, we merged six race categories into four. Thus, our sample size did not permit in-depth examination of racial and ethnic minority groups. Another limitation includes that Prolific does not consider ethnicity for representative samples; therefore, our sample was not representative based on ethnicity, only race. To mitigate bias, future health informatics recruitment efforts should prioritize diversity through approaches like community engagement and improved technology accessibility.

## Conclusion

Concerns expressed by research participants, especially participants from underrepresented racial groups, underscore the necessity of addressing participant perspectives to ensure that data sharing practices align with patient preferences. Prioritizing participant preferences for data sharing will prevent unintended barriers to recruitment and participation in research. Recognizing the varying levels of comfort participants have in sharing different types of sensitive data with external entities, it is vital to explore alternatives to broad consent, to ensure greater comfort and autonomy in deciding how data are shared.

## Supporting information

S1 Checklist Standards for Reporting Qualitative Research (SRQR)*. http://www.equator-network.org/reporting-guidelines/srqr/.
(PDF)

S1 File Supplementary methods. (DOCX)

S1 Table Prolific sample recruited by state in the US. (PDF)
