# Supplementary material for: Public perspectives on increased data sharing in health research in the context of the 2023 National Institutes of Health Data Sharing Policy
Source: PLoS One. 2024 Aug 28;19(8):e0309161. doi: 10.1371/journal.pone.0309161 (PMC11357082; doi:10.1371/journal.pone.0309161)
Supplement: S1 File — (DOCX) [file pone.0309161.s002.docx]

**S1: Supplementary Methods**

We conducted a survey of the general public using Prolific, an independent online recruitment platform to recruit a US representative sample. Prolific consists of an international sample of verified users who are willing to participate in research studies and facilitates a matching process between researchers and users for rapid recruitment and compensation for participating. Prolific uses US census data from the US Census Bureau to divide the sample into subgroups with the same proportions as the national population by age, gender, and race. The questions were developed through alliteration review and expert input from physicians and nurse-scientists. The survey was pilot tested with 10 members of the general public for clarity and length, and revised accordingly. The survey was conducted in English.

The survey questions and flow are provided below. The horizontal lines represent the blocking of the survey.


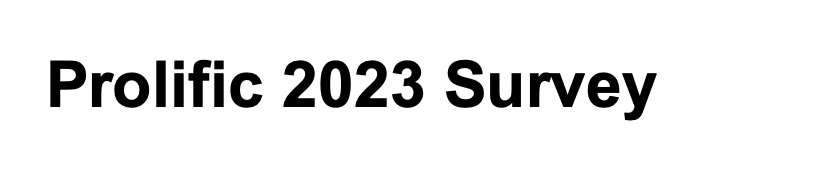


Survey Flow

**Start of Block: Introduction**

PID What is your Prolific ID?  
*Please note that this response should auto-fill with the correct ID. If not, please copy and paste your ID.* 

_______________________________________

NEXT Thank you for your interest in participating in this study. For more information, please download our [Information Sheet](https://cumc.co1.qualtrics.com/CP/File.php?F=F_1XQF3Zp9RkMbh4i).

- I agree to participate in this study.  (1)
- I do not agree to participate in this study.  (2)

________________________________________

INSTR_2 In some research studies, an individual's anonymous data is shared with others outside the research team. Anonymous data contains no direct personal identifiers of the participant. 
 
This is an example of data at the **individual** **level**.


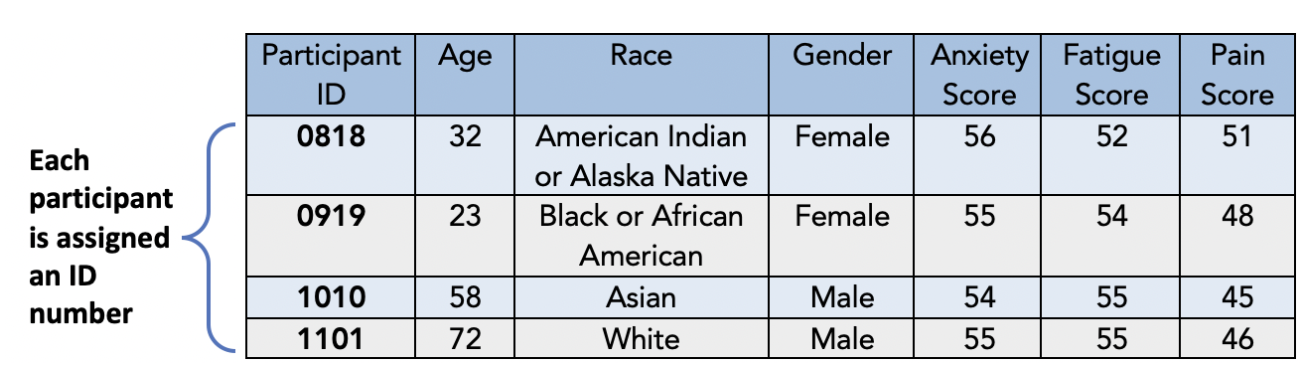


SHARE_Indiv_1 When I participate in research studies about my health, I want to share my data with...*(Select all that apply)*

- Health technology companies  (1)
- Health policy institutions  (2)
- Public health community organizations  (3)
- The public for other researchers to access  (4)
- On my social media  (5)
- Private foundations  (6)
- Other  (7) ______
- ⊗I do not want to share my individual results  (8)

______________________________________

SHARE_M_Indiv What kind of personal health data would you share with the following groups, if any? Check all that apply. **The following groups would know it is your data.** 

|  | Biological data (e.g., sensor, x-ray) (1) | Genetic data  (2) | Clinical data - (heart rate, blood pressure) (3) | Mental health (4) | Sexual health and/or fertility (5) | Imaging data (e.g., MRI, ultrasound) (6) | Consumer generated data (e.g. fitness tracker, quality of life) (7) | I would not share my data (8) |
| --- | --- | --- | --- | --- | --- | --- | --- | --- |
| Chosen family members (1) |  |  |  |  |  |  |  |  |
| Chosen friends (2) |  |  |  |  |  |  |  |  |
| Your doctors and nurses (3) |  |  |  |  |  |  |  |  |
| Other health care providers (4) |  |  |  |  |  |  |  |  |

_______________________________________

 SHARE_G_1 In some research studies, combined group results are shared with others outside the research team. 
 
 This is an example of **combined group** results.


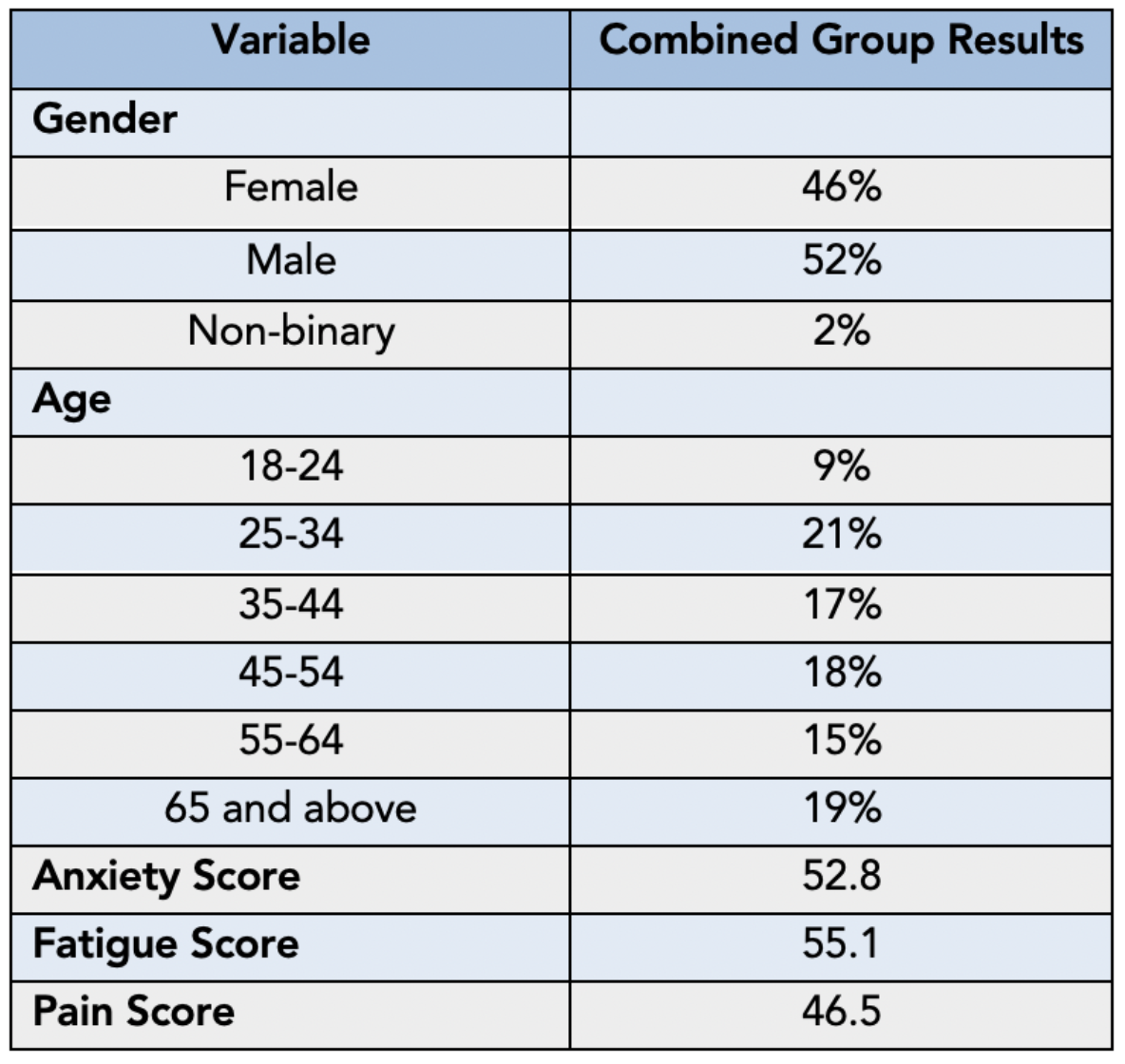


SHARE_G_2 When I participate in research studies about my health, I want the option to share my **combined group results** with... *(Select all that apply)*

- Health technology companies  (1)
- Health policy institutions  (2)
- Public health community organizations  (3)
- The public for other researchers to access and analyze  (4)
- On my social media  (5)
- Private foundations  (6)
- ⊗I do not want to share my combined group results  (7)
- Other  (8) ________
- ⊗I do not want to share my individual results  (9)

_______________________________________

INSTR_4


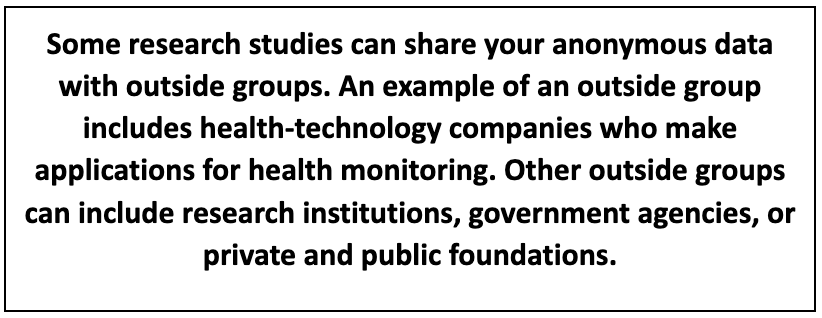


- I have read the statement above.  (1)

**Secondary_Matrix** What kinds of anonymous health data would you share with the following groups, if any? Check all that apply.

|  | Biological data (e.g., sensor, x-ray) (1) | Genetic data (2) | Clinical data - (heart rate, blood pressure) (3) | Mental health (4) | Sexual health and/or fertility (5) | Imaging data (e.g., MRI, ultrasound) (6) | Consumer generated data (e.g. fitness tracker, quality of life) (7) | I would not share my data (8) |
| --- | --- | --- | --- | --- | --- | --- | --- | --- |
| Health technology companies (1) |  |  |  |  |  |  |  |  |
| Health policy institutions (2) |  |  |  |  |  |  |  |  |
| Public health community organizations (3) |  |  |  |  |  |  |  |  |
| Public platforms for researchers to access (4) |  |  |  |  |  |  |  |  |
| Private foundations (5) |  |  |  |  |  |  |  |  |

_______________________________________

INSTR_6


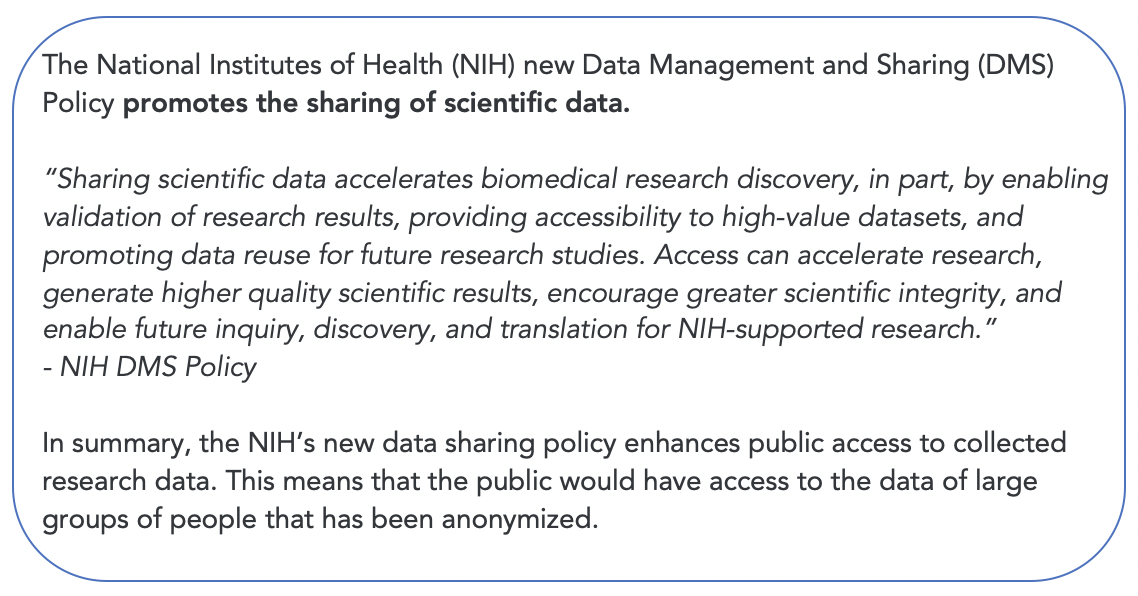


- I have read the statement above (1)

Agree_NIH Do you agree or disagree with the NIH's new efforts to make research data collected about you more accessible to the scientific community and the public?

- Strongly disagree  (1)
- Somewhat disagree  (2)
- Neither agree nor disagree  (3)
- Somewhat agree  (4)
- Strongly agree  (5)

Willingness_NIH Does the NIH's new efforts change **your willingness to participate** in a research study?

- Strongly increase my willingness to participate  (1)
- Increase my willingness to participate  (2)
- No change in my willingness to participate  (3)
- Decrease my willingness to participate  (4)
- Strongly decrease my willingness to participate  (5)

Comment_NIH Please provide any comments you have about the new NIH DMS policy.

_______________________________________

INSTR_7 **The federal government asks us to collect the following information to make sure that our results include everyone.**

DEM_Gender What is your current gender?

- Male  (1)
- Female  (2)
- Non-binary  (3)
- Transgender  (4)
- I use a different term  (5) ______
- Don't know  (6)
- Prefer not to answer  (7)

DEM_AGE What is your age?

_________________

DEM_RACE Which race best describes you?

- American Indian or Alaska Native  (1)
- Asian  (2)
- Black or African American  (3)
- Native Hawaiian or Other Pacific Islander  (4)
- White  (5)
- Multi-race  (6)
- Other (Describe below)  (7) ______

DEM_ETH Are you of Hispanic, Latino, or Spanish origin?

- Yes  (1)
- No  (2)
- Unsure/prefer not to answer  (3)

DEM_ORG What is your country of origin?

___________________

DEM_LANG What was the primary language spoken in your home?

- English  (1)
- Mandarin  (2)
- Spanish  (3)
- Other (Describe below)  (4) _________

DEM_FIN Do you feel you have enough financial resources to make ends meet?

- More than enough  (1)
- Enough  (2)
- Not enough  (3)

DEM_EDU What is the highest level of education you completed?

- Did not complete high school or receive GED  (1)
- High school or GED  (2)
- Some college or Associates degree (for example: AA, AS)  (3)
- Bachelor’s degree (for example: BA. BS)  (4)
- Master’s degree (for example: MA, MS, MEng, MEd, MSW, MBA)  (5)
- Professional degree beyond bachelor’s degree (for example: MD, DDS, DVM, LLB, JD)  (6)
- Doctorate degree (for example, PhD, EdD)  (7)

SURVEY_END **PLEASE NOTE**: You are about to submit this survey. Please review your answers before clicking next to submit.

_______________________________________
