# Supplementary material for: Public perspectives on increased data sharing in health research in the context of the 2023 National Institutes of Health Data Sharing Policy
Source: PLoS One. 2024 Aug 28;19(8):e0309161. doi: 10.1371/journal.pone.0309161 (PMC11357082; doi:10.1371/journal.pone.0309161)
Supplement: S1 Table — (PDF) [file pone.0309161.s003.pdf]

Supplementary Table 1. Prolific sample recruited by state in the US

State	Count	State	Count
Alabama	10	Nebraska	1
Arizona	16	Nevada	10
Arkansas	3	New Hampshire	2
California	61	New Jersey	10
Colorado	14	New Mexico	8
Connecticut	6	New York	39
Delaware	1	North Carolina	27
District of Columbia	5	North Dakota	2
Florida	43	Ohio	21
Georgia	25	Oklahoma	2
Idaho	5	Oregon	10
Illinois	20	Pennsylvania	25
Indiana	11	Rhode Island	6
Iowa	6	South Carolina	11
Kansas	3	South Dakota	1
Kentucky	11	Tennessee	8
Louisiana	12	Texas	48
Maine	2	Utah	7
Maryland	13	Virgina	16
Massachusetts	14	Washington	10
Michigan	22	West Virginia	3
Minnesota	6	Wisconsin	6
Maine	0	Vermont	0
Mississippi	4	Hawaii	0
Missouri	16	Alaska	0
Montana	1		
